# Evaluation of Lower Limb Asymmetry Index Based on the 30-Second Skater Squat Functional Test in Young Men

**DOI:** 10.3390/jcm13144017

**Published:** 2024-07-10

**Authors:** Mateusz Kamiński, Anna Katarzyna Cygańska

**Affiliations:** Faculty of Rehabilitation, Józef Piłsudski University of Physical Education in Warsaw, Marymoncka 34, 00-968 Warszawa, Poland; anna.cyganska@awf.edu.pl

**Keywords:** skater squat, limb asymmetry index, muscle strength, task performance, return to sport

## Abstract

**Introduction:** Physical performance tests (PPTs) are used for the pre-season evaluation of athletes and to monitor and control the rehabilitation process. PPTs include single-leg jumps, single-leg squats, and balance tests. One of the physical fitness tests is the skater squat test. The 30 s skater squat functional test (SSFT) is used as one of the tests to assess fitness and symmetry in the lower limbs. The present study aimed to calculate and compare the asymmetry index using the 30 s skater squat functional test, the single-leg distance jump test, and the isometric measurement of knee joint extensor strength. **Materials and Methods:** The study examined 25 men aged 23 ± 3.17 years. The study used the 30 s SSFT, the single-leg long jump test (SLLJT), and an isometric dynamometer test to measure peak moment of force values for extensors of the knee using the JBA Zbigniew Staniak^®^ measuring station (“JBA” Zb. Staniak, Poland). The statistical analysis of functional test results and iso-metric dynamometry results was based on correlation analysis. **Results:** There was a moderate correlation between 30 s SSFT and SLLJT (r = 0.540), and between SLLJT and measurements of peak moment of force of the knee joint extensors (r = 0.533). **Conclusions:** The asymmetry index calculated based on functional tests and peak moment of force of the knee extensors in a group of young men should not be used interchangeably. The asymmetry index calculated from the 30 s skater squat functional test detects greater differences in knee extensor strength than the ASI index calculated from the single-leg long jump test in a group of young male athletes. The practical significance of this study was that its results could play an important role in the training process and monitoring the return to sports after a possible injury.

## 1. Introduction

Physical performance tests (PPTs) are commonly used by physiotherapists to assess lower limb muscle function after injury, to monitor the progress of treatment and rehabilitation, for screening, or to assess the fitness of athletes of various skill levels throughout pre-season preparation [1,2,3]. The advantages of PPTs include easy protocols requiring no specialized knowledge, quick implementation, and being able to perform the test without expensive measurement equipment [1].

Isokinetic dynamometry is considered the gold standard for assessing the value of extensor force moments developed at the knee joint [4]. Similar sensitivity in detecting asymmetry of knee joint extensor force moments using isometric dynamometry at 90 degrees, and at 30 degrees of lower leg flexion at the knee joint, has been shown compared to isokinetic testing [5]. The vast majority of clinicians and trainers do not have access to this equipment, so PTTs are an alternative for assessing and monitoring athletes’ performance, as well as their recovery from injury [6]. 

An asymmetry index (ASI) based on PPT allows for the assessment of the progress of treatment, readiness to return to activity and sports participation, and athletic performance in people following surgical procedures or injuries to the lower limbs. LSI and ASI are most commonly used to evaluate the knee joint [7]. The literature identifies 12 different formulas for calculating the indices of symmetry and/or asymmetry, most of which require indicating the stronger limb. The four formulas allow for the determination of the stronger or weaker limb, but they yield different values, making it impossible to compare the results. When choosing a formula for calculating the LSI or ASI, it is important to consider the nature of the task, i.e., if it is unilateral or bilateral [8,9]. The $\frac{\left(A\;-\;B\right)}{Max\;\left(A,B\right)}\times 100$ (with A meaning left limb value and B meaning right limb value) formula is recommended for the evaluation of asymmetry based on unilateral tests because it involves normalization of the absolute difference to the value of the stronger limb [8].

It is important to distinguish between intra-limb asymmetry, i.e., different results for the same limb in the same test, and inter-limb asymmetry, and, therefore, different results for the right and left limbs in the same test [9,10]. 

Many studies that analyzed indices of symmetry and asymmetry have used the single-leg long jump test (SLLJT) [1,11]. Isokinetic dynamometers and isometric dynamometers that take into account the joint flexion angle are used to study the symmetry and asymmetry of the values of the peak moment of force of the knee joint extensors [7,11]. Objective testing of knee extensor strength without specialized equipment available only in research laboratories is a major challenge for clinicians and coaches. Clinical evaluation is increasingly using the 30 s skater squat functional test (30 s SSFT) to calculate LSI or ASI. One article presented the technique of performing the skater squat functional test and its progressions and regressions [12]. There are studies available that examine the correlation of the asymmetry, dynamic strength, and isometric force of lower limbs with different functional tests [11,13,14,15,16]. No studies comparing LSI/ASI based on 30 s SSFT or another timed squat test with LSI/ASI values based on muscle strength measured using isometric dynamometry were found in the available literature. Our hypothesis of the study was that evaluating limb asymmetry between right and left knee extensors according to the 30 s SSFT can successfully prove lower limb muscle weakness. The purpose of this study was to calculate and compare the asymmetry index using the 30 s skater squat functional test, the single-leg long jump test, and with isometric measurement of knee extensor strength. With this study, the utility of another functional test to monitor progress in the process of training as well as rehabilitation was evaluated.

## 2. Material and Methods

The research received approval from the Senate’s Research Bioethics Committee (SKE 01-511/2022) on 10 February 2023. All participants gave informed written consent to participate in the study after they were informed about the purpose and procedure of the experiment and the possibility of withdrawal from the study. 

### 2.1. Participants

The target population of the study is young, physically active adults, and twenty-seven people were enrolled in the study, of whom two were excluded (one due to limited range of motion in the ankle joint, and the other due to an ankle injury 3 weeks before the date of the study). Twenty-five men aged 23 ± 3.17 years old, with a body height 182 ± 7.49 cm, body weight 79 ± 9.43 kg and a BMI 24 ± 2.14, were studied. The study group was recruited on a convenience sampling basis. Among the participants, all participated in regular physical activity (weight training and/or other sports for more than 6 months, a minimum of 2 times a week). Inclusion criteria for the study were age between 18 and 35 years, consent to participate in the study, no weight training for lower limbs 72 h before the study, no medical contraindications to exercise, no musculoskeletal injuries in the lower limbs and trunk (within the last 3 months: fractures, sprains, dislocations, or mechanical instability of the ankle, knee, hip, or trunk). Exclusion criteria were female gender, current musculoskeletal injuries to the lower limb or trunk (within the past 3 months: fractures, sprains, dislocations, or mechanical instability of the ankle, knee, hip, or trunk), reported femoroacetabular impingement (FAI), and heel detachment during the skater squat test. 

### 2.2. Procedures

Before lower limb asymmetry measurements, anthropometric measurements and a 5 min warm-up (conducted under the supervision of a personal trainer with 2 years of professional experience) were performed. The warm-up was developed based on the literature and the clinical experience of the authors of this study and physiotherapist and consisted of A-skips over 20 m, C-skips over 20 m, 20 bodyweight squats, and 10 hops [17,18]. As a form of familiarization with the test (familiarization session) [19], two sets of skater squats and single-leg long jump test were performed, with five repetitions of each exercise (with a one-minute break between sets). The familiarization session was preceded by playing the author’s video of the correct execution of the tests. Additional verbal instructions were given if the participant significantly modified the performance of the test. Then, the measurements proper were carried out.

### 2.3. Measurements

**Figure 1:**
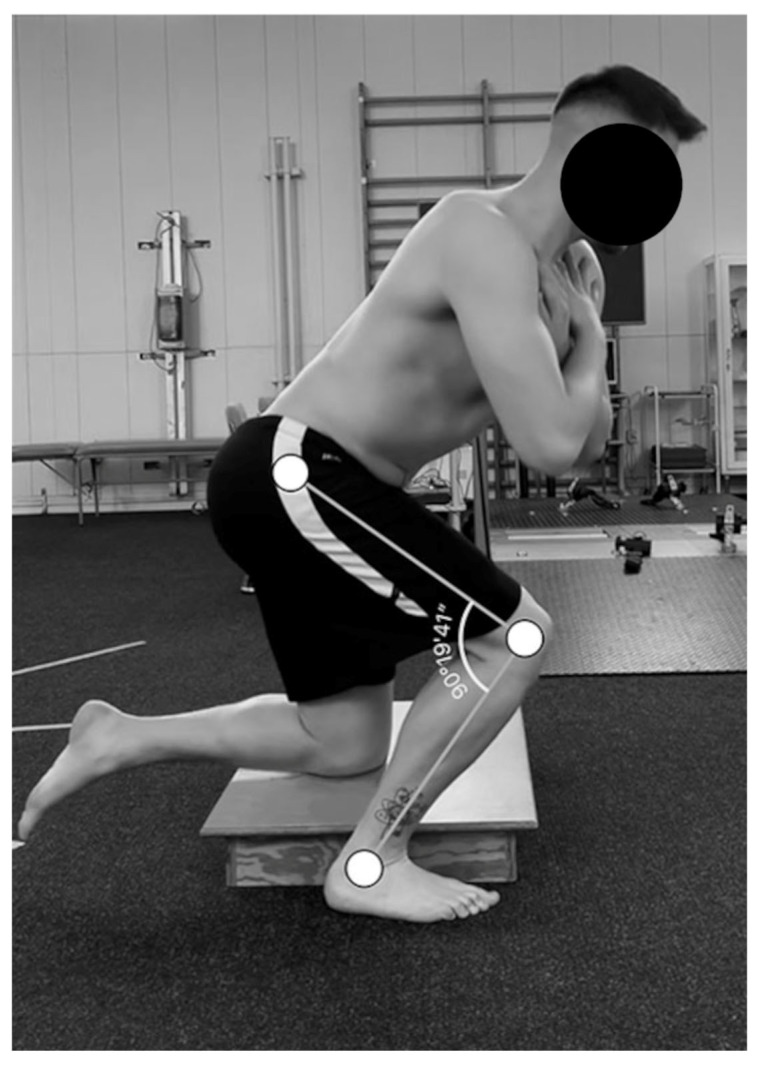
View in the sagittal plane, while performing the 30 s skater squat functional test (30 s SSFT). Source: own material.

The measurement using the 30 s skater squat functional test (30 s SSFT) was as follows: withe foot of the supporting limb was parallel to the sagittal plane, the non-tested limb should be flexed at the knee joint and during the squat, and the tibial tuberosity touched the step (with adjustable height), so that the angle of flexion at the knee joint tested was 90 degrees (measurement was performed using a Saehan 15 cm goniometer (SAEHAN, GripTM, Rulong, Belgium). Furthermore, one should pay attention to the adherence of the heel of the supporting limb to the ground and the lack of contact between the foot of the non-supporting limb and the ground [20]. The correct positioning of the limbs and step during the 30 s SSFT is illustrated in Figure 1. The verbal instruction before the test was as follows: “Do as many repetitions as possible in 30 s. Do not touch the ground with the foot of the non-tested limb. Do not detach the heel of the tested limb.” With the “Stop” command, time measurement was stopped, followed by a 3 min break before the next test. The procedure was recorded for later verification of the number of repetitions.

**Figure 2:**
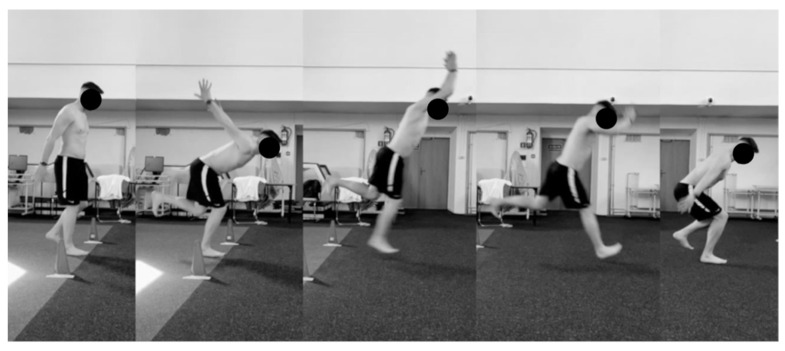
The correct technique for performing the single-leg long jump test (SLLJT). Source: own materials.

The single-leg long jump test (SLLJT) was carried out as follows: the front of the shoe was placed on the starting line and additional verbal instruction was given: “Perform the longest possible one-legged jump, land on the take-off leg, and maintain balance for 3 s. Don’t prop yourself up after you land.” The distance achieved in each test was marked with a marker, measured from the starting line to the back of the heel. The test included two correctly performed tests, of which the result with the higher value was used for later analysis. In the case of three incorrectly performed jumps in a row, the test was not scored, the measurement was not taken into account, and the test set had to be repeated after a break (not on the same day). There was no such case in this experiment. Failure to pass the test occurred in the event of loss of balance within the first 3 s after landing and propping up within the first 3 s with the non-tested lower limb or the upper limb [21]. The correct technique for performing the single-leg long jump test is shown in Figure 2.

**Figure 3:**
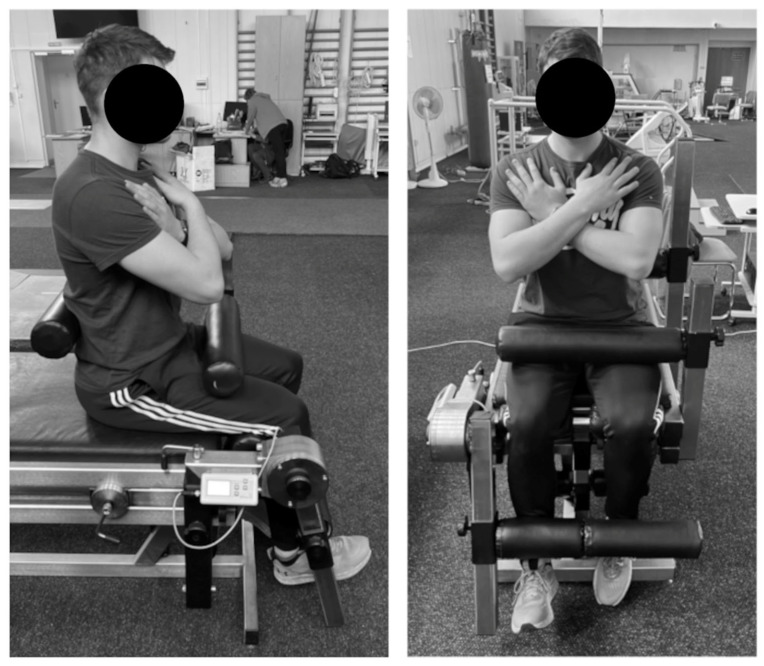
The position for measuring the peak moment of force of the knee joint extensors in isometric condition. Source: own materials.

The peak moment of force of the knee joint extensors was applied according to the maximal voluntary contraction method on a JBA Zbigniew Staniak^®^ measuring station (“JBA” Zb. Staniak, Warsaw, Poland) [22,23]. The peak moment of force of the knee joint extensors was measured in static measurements on a TBK3 stand designed by JBA Zb. Staniak. The parameter has a measurement range of 1200 Nm with a maximum measurement error of 1%. The vertical design ensures the optimal alignment of the trunk, knee, and hip joints in the seated position (the standard used in biomechanics laboratories). The axis of torque measurement is parallel to the axis of rotation of the joint. In the measurements, the maximum energy is delivered in 1.5–3.0 s. The maximum of the three highest torque values is recorded as the final measurement result [22,23]. Measurements were taken in a sitting position on a TBK3 JB Staniak stand, with stabilization on the lumbar spine and thighs. The upper limbs were crossed on the chest. The axis of the torque head was aligned with the axis of rotation of the knee joint. The resistance roller (gauge) was placed on the anterior surface of the lower leg above the talocrural joint. The position for measuring the peak moment of force of the knee joint extensors in isometric condition is shown in Figure 3.

### 2.4. Statistics

Statistical analysis was performed using Statistica 13.3 (TIBCO Software, Palo Alto, CA, USA). Descriptive statistics were used to characterize the study participants by calculating mean values and their standard deviations. The normality of the distributions was verified using the Shapiro–Wilk test, and all group data had normal distributions. Using a simple linear correlation (Person’s r), the relationship between 30 s SSFT, single-leg long jump test, and knee extensor strength values was calculated using all the variables together for the right and left lower limbs. A simple linear correlation (Person’s r) was used to test whether there was a reciprocal relationship between the ASI calculated from functional tests and the values of knee extensor strength. The statistical significance of differences was set at *p* < 0.05. The correlation coefficient (r) was interpreted as follows, based on the classification described by J. Cohen: 0–0.4 (weak), 0.4–0.7 (moderate), and 0.7–1.0 (strong) [24]. The ASI index was calculated from the formula proposed by Parkinson et al. [7] ASI = $\frac{\left(A\;-\;B\right)}{Max\;\left(A,B\right)}\times 100$, based on the number of repetitions in the 30 s SSFT, with A meaning the number of repetitions for the left lower limb and B meaning the number of repetitions for the right lower limb; based on the best distance obtained in the SLLJT, with A meaning the jump distance on the left lower limb and B on the right; and based on the highest value from three measurements of the peak moment of force of the knee joint extensors, with A denoting the measurement value for the left lower limb and B for the right. 

## 3. Results

**Figure 4:**
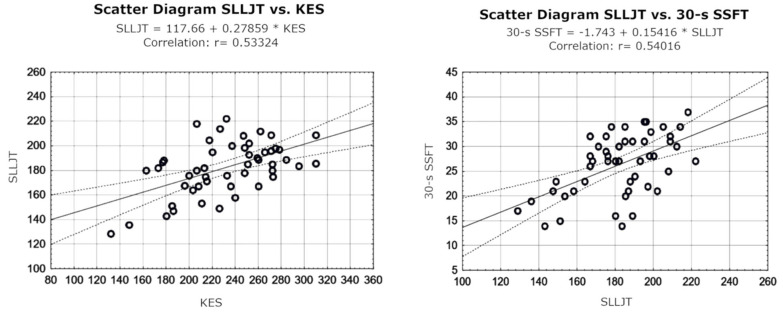
Scatter diagrams for the correlation coefficient (Persona’s r) of functional tests and peak moment of force of the knee joint extensors.

Analysis of the relationships between the results of 30 s skater squat functional test (30 s SSFT) (n = 50; 26.34 ± 6.11 reps; for right and left lower limb, respectively: n = 25; 26.56 ± 5.81 reps and n = 25; 26.12 ± 6.50 reps), single-leg long jump test (SLLJT) (n = 50; 182.17 ± 21.41 cm; for right and left lower limb, respectively: n = 25, 183.54 ± 20.56 cm and n = 25, 180.80 ± 22.56 cm) and values of the strength of knee extensors (KES) (n = 50; 231.56 ± 40.98 Nm; for right and left lower limb, respectively: n = 25 237.44 ± 42.63 Nm and n = 25, 225.68 ± 39.23 Nm) showed a moderate correlation (r = 0.540) between 30 s TFSS and SLLJT, and a moderate correlation (r = 0.533) between SLLJT and KES. Detailed results are shown Table 1 and Figure 4.

**Figure 5:**
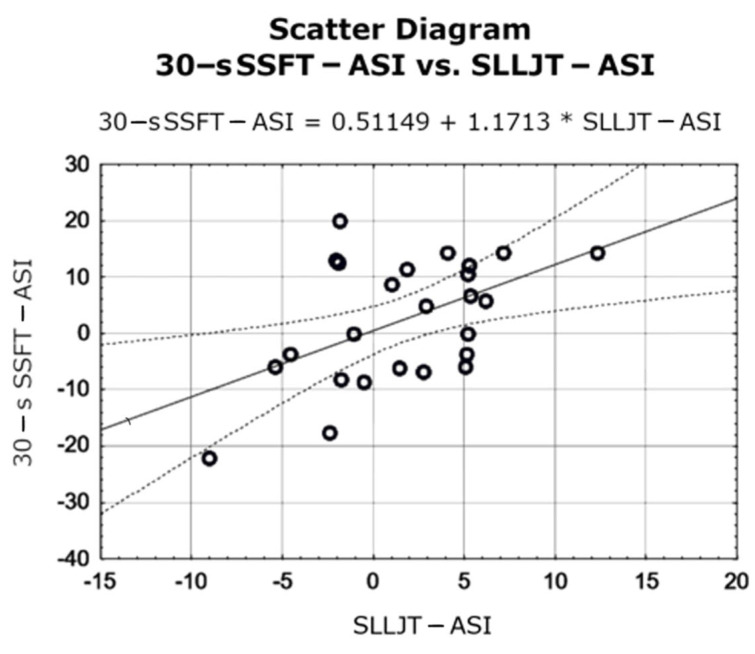
Scatters diagrams for the ASI index correlation coefficient calculated from functional tests and peak moment of force of the knee joint extensors.

Statistical analysis of the relationship between the asymmetry index (ASI) values calculated from 30 s SSFT (2.38 ± 10.98), SLLJT (1.59 ± 4.70), and KES (4.56 ± 8.84) showed a moderate correlation (r = 0.501) between the ASI calculated from the 30 s SSFT test and SLLJT. Detailed results are shown in Table 2 and Figure 5.

The value of the ASI calculated from the functional tests was lower compared to the value of the ASI calculated using the values in the strength of knee extensors. The ASI value calculated based on SLLJT was lower compared to the ASI calculated using 30 s SSFT. Table 3 shows the mean values and standard deviations of the ASI calculated from the functional tests and KES.

## 4. Discussion

The present study is one the first to address skater squat problems and the first in the world to calculate the ASI index from a timed single-leg squat test. To date, there have been no studies comparing the ASI 30 s SSFT index with other functional tests or the peak moment of force of the knee joint extensors. The ASI index for the 30 s SSFT test was shown to detect larger average asymmetries than SLLJT.

There was a moderate correlation between 30 s SSFT and SLLJT and between SLLJT and strength of knee extensors. Furthermore, there was no correlation between 30 s SSFT and strength of knee extensors (shown in the results section, Table 1). No articles were found in the literature to examine the relationships between the timed single-leg squat test and the values of peak moment of force of the knee joint extensors. Only two reports were available to examine the relationships between SLS depth and the strength of knee extensors measured with an isokinetic dynamometer. Batty et al. [15] found a weak linear correlation between squat depth and the strength of knee extensors, while Östenberg et al. [25] found no relationship between the strength of knee extensors and squat depth. Reports on the relationships of SLLJT with objective measurements of knee extensor strength in healthy populations are inconsistent. Östenberg et al. [25] and Vassis et al. [26] showed no relationship between the distance in the SLLJT test and the strength of knee extensors. Other researchers have shown moderate to strong correlations between SLLJT and isokinetic measurements of the strength of knee extensors [16,27,28]. In another study, Vassis et al. [29] found that the strength of knee extensors measured under isokinetic conditions was a significant predictor of TSJNO, explaining 40.4% of the variance for 180°/s and 45.3% of the variance for 60°/s angular velocities. The results of the study should be interpreted with caution, as results of about 40% do not explain 100% of the variation and do not explain more than half of the variance. This means that the strength of knee extensors measured with an isokinetic dynamometer was not the only variable affecting SLLJT test results. Other causes that may affect test results should be considered, e.g., neuromuscular control. It is claimed that no other simple measurement is as strongly related to strength of knee extensors as the SLLJT. One study was found describing squat depth as a predictor of knee strength in patients 6 and 12 months after ACL reconstruction. Batty et al. [15] demonstrated that an LSI of <90% (ASI > 10%) between the operated and non-operated limb is a predictor of an LSI < 90% (ASI > 10%) of the strength of knee extensors measured using an isokinetic dynamometer. The sensitivity of this test was determined to be 34.7 at 6 months and 17.5 at 12 months following the ACL reconstruction. Specificity was defined as 80 at 6 months and 79.1 at 12 months following the ACL reconstruction. There are many indications that maximum arbitrary flexion angle returns to symmetry >90% faster than the strength of knee extensors. Due to the high specificity of the maximum flexion test during the single-leg squat, it has clinical value and can complement the functional evaluation of patients after ACL reconstruction. 

There was a statistically significant moderate correlation between the ASI for lower limbs calculated from the 30 s SSFT and the SLLJT. There was no statistically significant correlation between the ASI calculated from the functional tests results and the value of peak moment of force of the knee joint extensors. There are discrepancies in the literature regarding the methods of calculating LSI and ASI indices, with the antinomic (apparently correct, but leading to contradictions) terms of symmetry and asymmetry being used interchangeably, which obliges the reader to be careful and vigilant in interpreting the results. This is because 0% asymmetry is equivalent to 100% symmetry, which means no asymmetry and, therefore, full symmetry [8]. Another problem that arises when interpreting the results of studies on the symmetry and asymmetry of lower limbs is the different methods of calculating their indices. Twelve different formulas for calculating symmetry and asymmetry indices have been found in the literature, some of which require choosing the stronger lower limb in advance, which does not work when testing a healthy population [30,31,32,33,34,35,36]. The asymmetry index *ASI* = $\frac{\left(A\;-\;B\right)}{Max\;\left(A,B\right)}\times 100$, which was used in the present study and does not require a prior reference to a stronger limb, has also been used in previous studies, [37,38]. In this study, ASI was calculated for the first time using a 30 s single-leg squat test, but there are no studies with could be used for comparison. Many authors suggest that functional tests underestimate the level of asymmetry (overestimate the level of symmetry) compared to those using dynamometers [39,40]. In the present study, it was observed that the mean asymmetry results obtained from the functional tests were lower compared to the results of the peak moment of force of the knee joint extensors (ASI—SLLJT 1.6 ± 4.7, ASI—30 s SSFT 2.4 ± 11.0 and ASI SLLJT 4.6 ± 8.8), while the mean asymmetry scores obtained from the 30 s SSFT were higher than those obtained from the SLLJT test (ASI—SLLJT 1.6 ± 4.7 and ASI—30 s SSFT 2.4 ± 11.0). Available scientific evidence shows that an LSI > 90% calculated from three jump tests (including SLLJT) may be an insufficient criterion for safe participation in physical activity and competitive sports [41]. Based on the available scientific evidence and the clinical experience of physiotherapy specialists, physicians, and coaches, the current criteria for returning to participation in sports after injury include such aspects as no swelling, a full range of motion of the knee joint, no joint instability, and thigh circumference measured 15 cm above the patella not less than 1.5 cm compared to the non-operated lower limb. The symmetry index for the strength of knee extensors measured using an isokinetic dynamometer was above 90% (ASI < 10%), the scoring on the FMS test was 14 or higher, and a symmetry index of more than 90% was obtained in four jump tests and landing after a jump using the landing error scoring system [41]. 

It is important to emphasize the value of functional tests which aim to evaluate factors, such as muscle strength, joint stability, neuromuscular control, and overall health [42,43]. Given the need to develop the most effective methods of verification and precise criteria for return to sport after injury (e.g., ACL), it is reasonable to attempt to include the 30 s SSFT test as another criterion for return to sport. This test is quick to perform and provides additional information on the functional performance of the limb after reconstruction surgery. The list of requirements before returning to sport after an ACL injury is long and requires access to specialized and expensive equipment. In addition to the criteria of muscle strength and fitness for a safe return to full participation, the athlete must meet appropriate psychological criteria, and appropriate athlete management should be implemented to reduce risk factors for re-injury [44]. Meeting all the conditions on the list still does not eliminate the risk of re-injury and does not accurately identify patients at increased risk of ACL re-injury [45]. This shows how complex the topic of ACL reconstruction and subsequent rehabilitation is. New surgical techniques and rehabilitation protocols are being developed all the time for a safe return to activity and participation, but confirmation of their effectiveness requires randomized controlled clinical trials with a long follow-up period. 

The 30 s SSFT results obtained in the paper do not correlate with those of objective measurements, i.e., peak muscle force measured with isometric dynamometers. This does not mean that this test cannot be applied in practice. In order for the 30 s SSFT to find use among clinicians, using standardized procedures and adherence to them during implementation are required. It is also not suitable for all patients, as the movement task of the single-leg squat requires adequate motor coordination, muscle strength, and mobility in the peripheral joints of the ACC. The 30 s TFSS can find application in athletes and physically active people as one of the components of assessing overall fitness. Furthermore, the introduction of regular standardized testing in sports teams could help clinicians more accurately determine the function of a limb before injury. A major challenge for physiotherapists and coaches is to estimate the function of the lower limb before the injury. Sports differ in the specifics of their movement tasks, which can translate into greater or lesser levels of asymmetry. In sports with unilateral asymmetry, e.g.,: long jump, with athletes preferring one of the limbs during the run-up, asymmetry in knee extensor strength may be a natural adaptation to the sport [46]. Comparison of limb strength and functional performance after injury is fraught with errors. First, it is unclear which lower limb was stronger before the injury. Secondly, as a result of the break required for recovery, there is a high probability that the strength and function of the unused limb have deteriorated, which is the determinant when calculating the symmetry or asymmetry indices. Regular functional fitness tests, such as the 30 s SSFT and the cluster of jump tests, can serve as a baseline for the assessment of lower limb injuries. If possible, it is worth extending the testing of athletes with regular measurements of the peak muscle force in the lower limb.

This study assessed the utility of the 30 s SSFT as a functional test to monitor exercise and rehabilitation progress. Despite its novel approach to the research problem, the study has minor limitations that are worth pointing out and addressing in future research. First, measurements of the strength of knee extensors were assessed using an isometric dynamometer. In the literature, testing the strength of knee extensors with an isokinetic dynamometer is considered the gold standard. Second, a sample size analysis was not performed, as there have been no results of studies on 30 s SSFT to date. Third, it is worth including female participants in future studies, and assessing the impact of confounding variables within this group of participants. Another aspect worth including in future studies would be to perform an internal and external concordance analysis for the 30 s SSFT visual assessment performed by different researchers. The practical significance of this study was that its results could play an important role in the training process and monitoring the return to sports after a possible injury. 

## 5. Conclusions

The asymmetry index calculated based on functional tests and values of knee extensor strength in a group of young men should not be used interchangeably. The asymmetry index calculated from the 30 s skater squat functional test detects greater differences in knee extensor strength than the ASI index calculated from the single-leg long jump test in a group of young male athletes.

## Figures and Tables

**Table 1:** Correlation coefficient (Persona’s r) of functional tests and peak moment of force of the knee joint extensors.

VARIABLE	SLLJT	KES
30 s SSFT	0.540 *	0.241
SLLJT	−	0.533 *

* Significant correlation level *p* < 0.05; SLLJT—single-leg long jump test; 30 s SSFT—30 s skater squat functional test; KES—knee extensor strength.

**Table 2:** ASI index correlation coefficient calculated from the functional tests and peak moment of force of the knee joint extensors.

VARIABLE	SLLJT—ASI	KES—ASI
30 s SSFT—ASI	0.501 *	0.116
SLLJT—ASI	−	0.036

* Significant correlation level *p* < 0.05; SLLJT—ASI—single-leg long jump test asymmetry index; 30 s SSFT—ASI—30 s skater squat functional test asymmetry index; KES—ASI—strength of knee extensors asymmetry index.

**Table 3:** ASI calculated from the functional tests and peak moment of force of the knee joint extensors (M ± SD).

VARIABLE	Value n = 25
30 s SSFT—ASI	2.4 ± 11.0
SLLJT—ASI	1.6 ± 4.7
KES—ASI	4.6 ± 8.8

30 s SSFT—ASI—30 s skater squat functional test asymmetry index; SLLJT—ASI—single-leg long jump test asymmetry index; KES—ASI—strength of knee extensors asymmetry index.

## Data Availability

Dataset available on request from the authors.
